# Contamination of mononuclear cell suspensions obtained from cancer patients by the Böyum method.

**DOI:** 10.1038/bjc.1978.244

**Published:** 1978-10

**Authors:** G. A. Currie, D. W. Hedley, R. E. Nyholm, S. A. Taylor


*Br. J. Cancer* (1978) **38**, 555

***Short Communication***

### CONTAMINATION OF MONONUCLEAR CELL SUSPENSIONS OBTAINED FROM CANCER PATIENTS BY THE BÖYUM METHOD

G. A. CURRIE, D. W. HEDLEY, R. E. NYHOLM AND S. A. TAYLOR

*From the Department of Tumour Immunology, Chester Beatty Research Institute, And The Royal Marsden Hospital, Belmont, Sutton, Surrey*

Received 3 July 1978 Accepted 14 July 1978

ONE-STEP techniques for the isolation of lymphocytes by centrifugation of whole blood through mixtures of Ficoll and sodium metrizoate (Böyum, 1968) have the advantage of speed, simplicity and apparent efficiency. Mononuclear cell suspensions obtained by this procedure have been widely used for many types of assay, including the examination of lymphocyte sub-populations, lymphocyte responses to plant lectins, a variety of diagnostic cancer tests and the investigation of lymphocyte cytotoxicity.

The presence of monocytes in the so-called lymphocyte suspensions is now generally recognized, though the extent of this contamination is frequently underestimated or even disregarded, despite Zucker-Franklin's (1974) important warning.

In the course of examining a variety of functional assays of both lymphocytes and monocytes obtained by the Böyum method, we became concerned about the extent of "contamination" of mononuclear cell suspensions with cells other than lymphocytes, especially in samples obtained from cancer patients.

The isolation technique was exactly as described by Böyum (1968) except that we used a commercial preparation of Ficoll-sodium metrizoate (Lymphoprep, Nyegaard). Mononuclear cell suspensions (obtained from the interface) were prepared from defibrinated peripheral venous blood from 47 healthy age-matched normal donors, or from patients with histologically proven active malignant disease (either malignant melanoma (31) or breast carcinoma (45)). The final mononuclear cell suspensions when examined by high-power phase-contrast microscopy comprised relatively uniform populations of small round cells. Samples from each cell suspension were air-dried onto clean glass slides, fixed in formol acetone and stained for non-specific esterase (NSE) and/or chloroacetate esterase (CAE) as described by Yam *et al.* (1971). The percentage positive cells for each stain was counted on at least 400 cells by conventional light microscopy.

*Non-specific esterase (NSE) positive cells.*—Monocytes in the cell suspensions were readily identified by the presence of dense and diffuse reddish-brown cytoplasmic staining. Under the incubation conditions used, T lymphocytes showed no diffuse staining. Furthermore, all the densely $NSE^+$ cells could be removed by passage through nylon wool or by removal of adherent cells in plastic culture flasks.

In normal donors the percentage of monocytes was $18{\cdot}6 \pm 9{\cdot}6$, and in cancer patients $18{\cdot}9 \pm 10{\cdot}2$. In other words there was a substantial population of monocytes in the cell suspensions, and although the standard deviations were large there was no significant difference in the extent of contamination between normal donors and cancer patients.

FIG.—Frequency-distribution histogram of the percentage CAE+ cells in so-called mononuclear-cell suspensions from normal donors (solid columns) and untreated cancer patients (hatched columns).

*Chloroacetate esterase (CAE) positive cells.*—This staining method is specific for cells of the granulocyte series and their precursors. CAE+ cells were readily detected by the presence of intense scarlet cytoplasmic staining.

The percentages of CAE+ cells are shown in the histogram in the Figure where it can be seen that high levels of contamination were frequently found in the cancer patients. By conventional Giemsa staining these cells were rarely mature granulocytes, but presented a range of morphological features characteristic of early cells in the myeloid series. However, conventional morphological criteria are difficult to apply to cells which have been isolated on Ficoll-Metrizoate. The patients who provided large numbers of CAE+ cells were mostly those with advance disease, but by standard haematological techniques they showed no evidence of leukoerythroblastic anaemia. This is an alarming finding, and we have tried to minimize this contamination by changing several of the experimental conditions for the centrifugation procedure. We have examined the effect of temperature, speed and duration of centrifugation, extent of dilution of the blood and geometry of the centrifuge tube.

None of these variables influenced the extent of CAE+ cell contamination, presumably because the positive cells (left-shifted granulocytes) are separating on the basis of density.

Since in our hands a substantial number of cancer patients provide cell suspensions in which lymphocytes are a minor subpopulation ($< 50\%$) this method of cell separation cannot, we suggest, be used without the most rigorous quality control. The routine use of simple enzyme cytochemical assays for NSE and CAE may help to avoid some of these pitfalls. The presence of large numbers of "left-shifted" granulocytes in cell suspensions from cancer patients may provide an explanation for many of the published differences between cancer patients and normal donors, such as reduced T-cell levels, reduced lectin responses and other lymphocyte abnormalities.

### REFERENCES

BÖYUM, A. (1968) Isolation of mononuclear cells and granulocytes from human blood. *Scand. J. Clin. Lab. Invest.*, **21**, 77.

YAM, L. T., LI, C. Y. & CROSBY, W. H. (1971) Cytochemical identification of monocytes and granulocytes *Am. J. Clin. Path.*, **55**, 283.

ZUCKER-FRANKLIN D. (1974) The percentage of monocytes among "Mononuclear" cell fractions obtained from normal human blood. *J. Immunol*, **112**, 234.


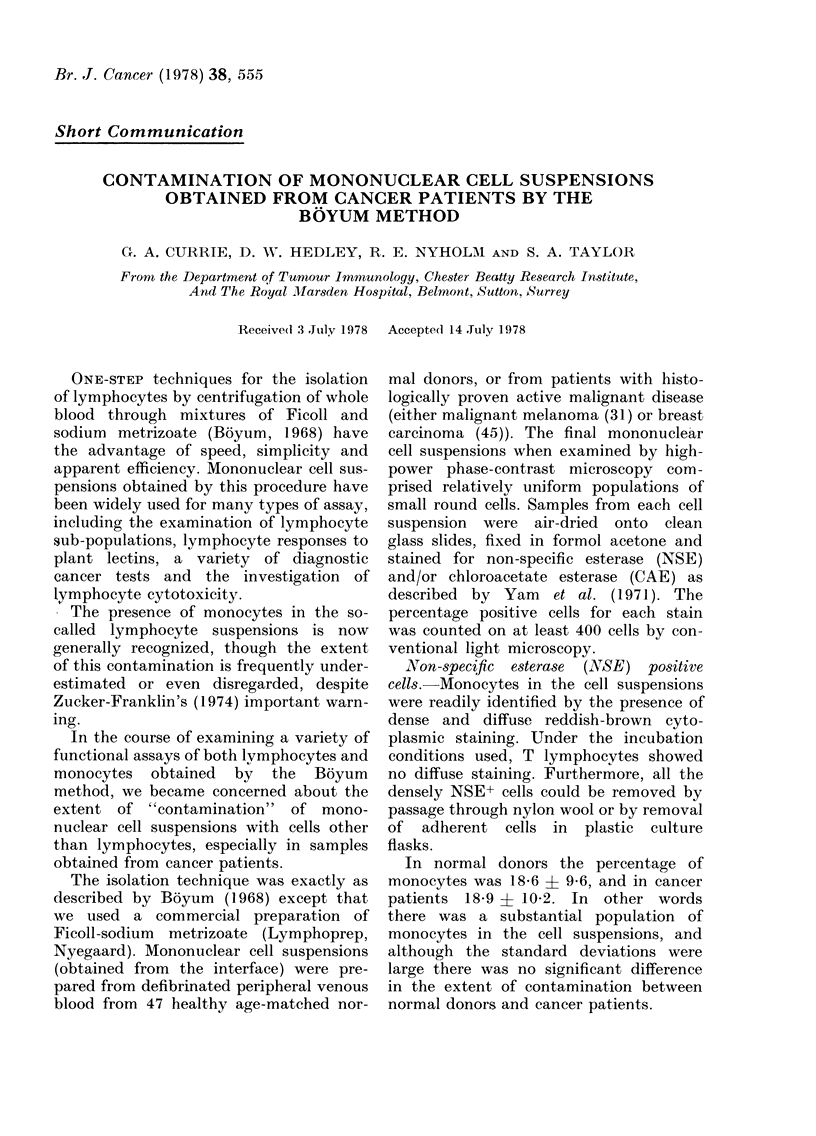

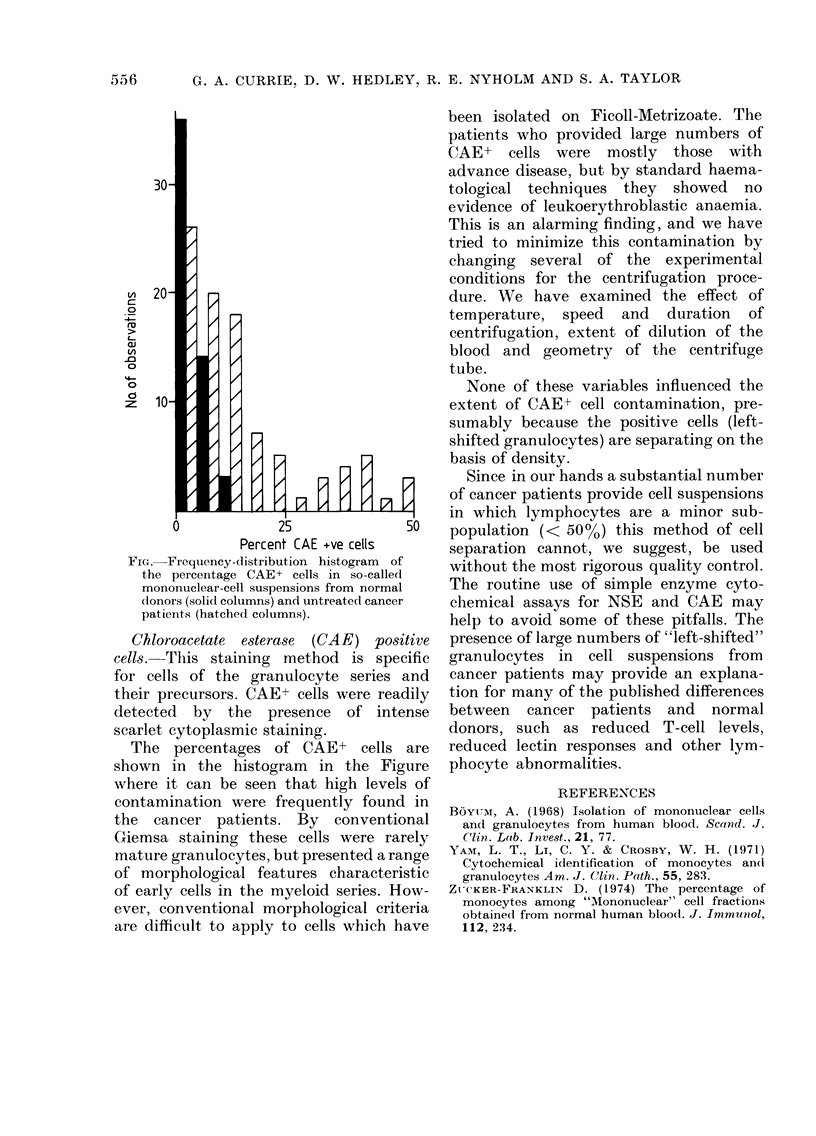


## References

[OCR_00192] Yam L. T., Li C. Y., Crosby W. H. (1971). Cytochemical identification of monocytes and granulocytes.. Am J Clin Pathol.

[OCR_00197] Zucker-Franklin D. (1974). The percentage of monocytes among "mononuclear" cell fractions obtained from normal human blood.. J Immunol.

